# How topology governs cation affinity: protonation and metal coordination in 1- and 9-azahomocubane

**DOI:** 10.1039/d5ra09156j

**Published:** 2026-02-06

**Authors:** Zahra Adeli, Morteza Rouhani, Bahareh Sadeghi

**Affiliations:** a Department of Chemistry, SR.C., Islamic Azad University Tehran Iran morteza.rouhani@iau.ac.ir; b Department of Chemistry, CT.C., Islamic Azad University Tehran Iran

## Abstract

The interaction of cationic species with aza-homocubanes provides a powerful platform for probing the effects of cage topology on electronic structure and noncovalent binding phenomena. Here, density functional theory (DFT) calculations were employed to investigate the complexation of 1-azahomocubane and 9-azahomocubane with H^+^, Li^+^, Na^+^, K^+^, Mg^2+^, and Ca^2+^. Optimized geometries reveal systematic trends in cation–nitrogen bond distances, with protonation yielding short covalent-type N–H^+^ interactions (∼1.0 Å), alkali metals displaying increasing separation with ionic radius (N–Li ≈ 1.9 Å, N–Na ≈ 2.3 Å, N–K ≈ 2.7 Å), and alkaline earth dications forming significantly shorter and stronger bonds than size-comparable alkali cations (N–Mg^2+^ ≈ 2.0 Å *vs.* N–Na ≈ 2.3 Å). Analysis of Hirshfeld charges confirmed substantial electron transfer from nitrogen to the bound cation, with the charge on N shifting from −0.134*e* in free 1-aza to +0.067*e* upon protonation, and from −0.163*e* in free 9-aza to +0.058*e* in its protonated form. For metal complexes, the nitrogen charges became even more positive, *e.g.*, −0.141*e* → +0.666*e* in (1-aza + Li)^+^ and −0.158*e* → +0.663*e* in (9-aza + Li)^+^, highlighting significant charge redistribution. The cation affinity (CA) and CB (cation basicity) indices quantified the stabilization: for instance, CA/CB values of 48.84/41.50 kcal mol^−1^ were obtained for (1-aza + Li)^+^ compared to 46.13/38.91 kcal mol^−1^ for (9-aza + Li)^+^, confirming stronger binding in 1-aza. The non-covalent interaction (NCI) isosurfaces and reduced density gradient (RDG) profiles revealed localized covalent-like interactions for protonated species, while alkali metals exhibited more diffuse electrostatic contacts, with dications displaying highly concentrated interaction regions. Collectively, the results reveal subtle but systematic differences between the two isomers, with 1-azahomocubane often exhibiting slightly shorter interaction distances and marginally enhanced stabilization relative to 9-azahomocubane, highlighting the influence of nitrogen topology on cation binding.

## Abstract 2

The interaction of cationic species with aza-homocubanes provides a powerful platform for probing the effects of cage topology on electronic structure and noncovalent binding phenomena.

## Graphical abstract

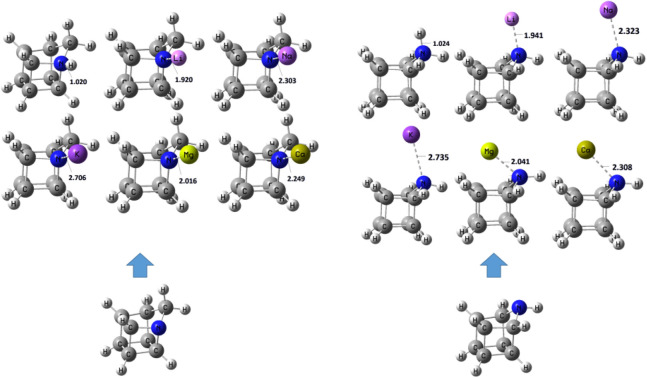

## Introduction

Cage-like hydrocarbons and their heteroatom analogues have long attracted attention owing to their unusual bonding, high strain energies, and unique electronic properties.^1–4^ Among these systems, homocubane derivatives represent a fascinating class of polycyclic molecules characterized by rigid frameworks and atypical bridgehead positions.^5,6^ The incorporation of heteroatoms such as nitrogen into these strained cages produces azahomocubanes, which exhibit distinct structural and electronic features compared with their hydrocarbon counterparts.^7–10^ Such modifications alter the reactivity and potential functional applications of these molecules, making them attractive candidates for fundamental studies as well as for possible use in supramolecular and materials chemistry.^11–13^ Nitrogen substitution in cage systems is particularly significant because the lone pair at the heteroatom provides a natural binding site for protons and metal cations.^14,15^ Proton affinity and cation coordination ability are key descriptors of the electronic characteristics of nitrogen heterocycles.^16–18^ In conventional acyclic or planar heteroaromatics, the relationship between structure and proton affinity is well established;^19,20^ however, in rigid, highly strained polycyclic environments, the accessibility, orientation, and electronic availability of the lone pair are strongly influenced by molecular topology.^21–23^ Consequently, the study of proton and metal ion binding in aza-cage molecules offers valuable insight into the interplay between geometry, electronic distribution, and noncovalent stabilization.^24–26^ Metal–nitrogen interactions are also of great importance in coordination chemistry, catalysis, and ion recognition.^27–29^ Alkali and alkaline earth cations, in particular, are fundamental in biological and chemical processes, and their selective recognition remains a central challenge in host–guest chemistry.^30,31^ Designing scaffolds that can effectively differentiate between cations of varying size and charge density is a longstanding goal in supramolecular chemistry.^32^ Polycyclic aza-frameworks, with their constrained geometries, offer a promising but underexplored platform for such investigations.^33^ These type studies have been successfully performed to diverse host–guest and cation–ligand systems, but their application to aza-homocubanes remains limited. In this context, the present work focuses on a comparative study of 1-azahomocubane (1-aza) and 9-azahomocubane (9-aza). These two isomers differ only in the position of the nitrogen atom, yet this seemingly subtle variation drastically alters the accessibility of the lone pair and the steric environment of the binding site. By systematically examining their interactions with H^+^, Li^+^, Na^+^, K^+^, Mg^2+^, and Ca^2+^, we aim to elucidate how molecular topology governs cation binding strength, electron density redistribution, and noncovalent stabilization. The integration of geometrical parameters, Hirshfeld charges, CA/CB indices, NCI isosurfaces, and RDG analyses provides a comprehensive picture of these interactions, offering insights not only into aza-homocubane chemistry but also into broader principles of ion recognition in strained nitrogen-containing frameworks.

## Computational methods

The molecular structure of 1-azahomocubane and 9-azahomocubane have been thoroughly optimized across B3LYP/6-311++G(d,p) computational level. The efficiency of this computational level for predicting properties and structure in cubane derivatives has been confirmed previously.^34,35^ Moreover, it was noted that incorporating dispersion corrections into the prevalent B3LYP methodology notably enhances conformity with experimental findings.^36^ Accordingly, the chemical structures of selected cations, along with their complexes with 1-azahomocubane and 9-azahomocubane, underwent thorough optimization utilizing the B3LYP-D3/6-311++G(d,p) level of theory.^37^ Cation affinity (CA) and cation basicity (CB) are defined as −Δ*H* and −Δ*G* for the interactions of the cations H^+^, Li^+^, Na^+^, K^+^, Mg^2+^, and Ca^2+^ with 1-azahomocubane and 9-azahomocubane, which for their obtaining, the frequency calculations were conducted in the gas phase at a temperature of 298 K. The affinity of a cation (CA) for a given azahomocubane (AHC) structure is quantitatively defined as the negative change in enthalpy (−Δ*H*) from the interaction expressed as follows:AHC + M^*n*+^ → (AHCM)^*n*+^

All computations were executed using Gaussian 16 software.^38^ The density of states (DOS) profiles were obtained using GaussSum software,^39^ and the non-covalent (NCI) isosurfaces and reduced density gradient (RDG) plots were performed using Multiwfn program code.^40^

## Results and discussion

**Fig. 1:**
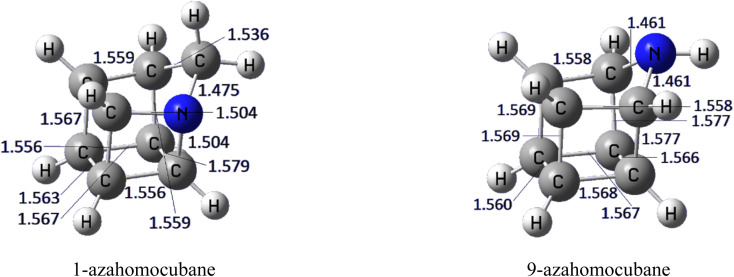
Optimized chemical structures of 1-azahomocubane and 9-azahomocubane.

Optimized molecular structures of 1-azahomocubane (1-aza) and 9-azahomocubane (9-aza) are shown in Fig. 1. The geometries of 1-azahomocubane and 9-azahomocubane reveal subtle but important structural variations arising from the position of the nitrogen atom in the homocubane skeleton. In 1-azahomocubane, the nitrogen atom is embedded within a bridgehead position, leading to stronger electronic perturbations of the cage framework due to the inherent strain at this site. This substitution induces noticeable bond length alternations around the nitrogen center and introduces additional angular strain within the polycyclic network. Conversely, in 9-azahomocubane, the nitrogen atom occupies a more peripheral site, where the steric congestion is less pronounced. As a result, the overall geometric distortion of the cage is reduced, preserving the near-symmetric cubane-like topology. These geometric characteristics indicate that the electronic consequences of nitrogen substitution are expected to be more pronounced in the 1-aza derivative, whereas the 9-aza system may retain greater electronic delocalization.

**Fig. 2:**
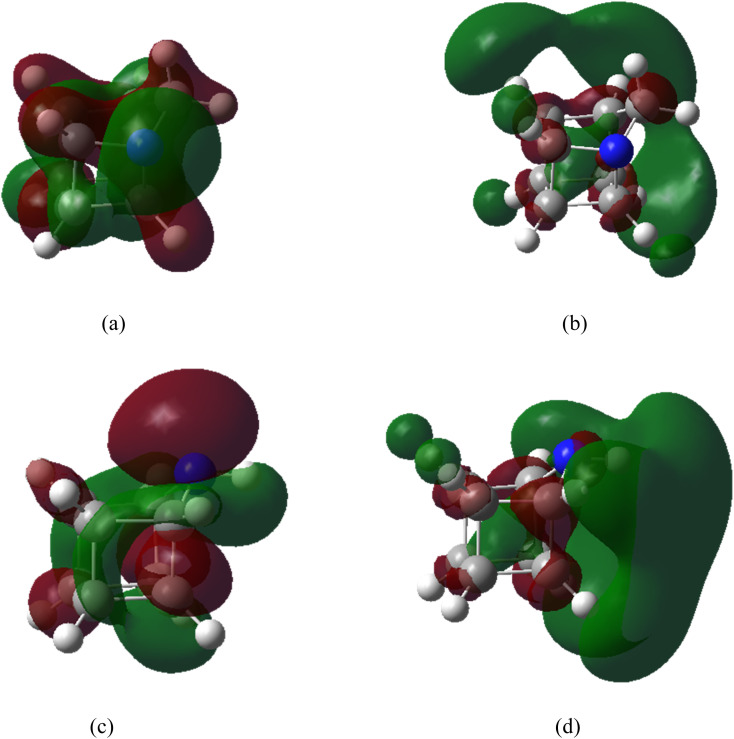
HOMO (a) and LUMO (b) of 1-azahomocubane, HOMO (c) and LUMO (d) of 9-azahomocubane (orbital isosurfaces are plotted at an isovalue of 0.02 a.u.).

The HOMO and LUMO profiles of 1-azahomocubane and 9-azahomocubane are demonstrated in Fig. 2. The frontier molecular orbital analysis provides deeper insight into the electronic structure and reactivity patterns of both aza-homocubanes. The frontier molecular orbitals of 1- and 9-azahomocubane are qualitatively similar in both spatial distribution and energy. In both isomers, the HOMO exhibits appreciable density around the nitrogen atom as well as neighboring carbon atoms, reflecting the involvement of the lone pair within the rigid cage framework. Any differences in orbital localization are subtle. Accordingly, the interaction trends discussed below cannot be rationalized on the basis of a single frontier orbital, but rather arise from cumulative, small changes across several occupied orbitals, as supported by the DOS analysis.

**Fig. 3:**
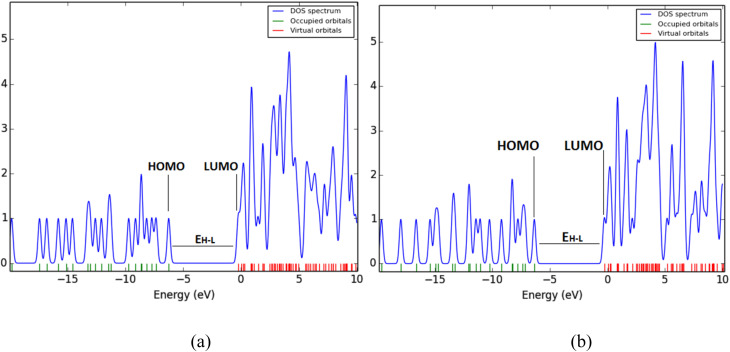
DOS diagrams of 1-azahomocubane (a) and 9-azahomocubane (b).

The total and projected density of states (DOS) diagrams (Fig. 3) reveal differences in the electronic structures of 1-azahomocubane and 9-azahomocubane. In both isomers, the occupied manifold is composed primarily of carbon σ-type states with significant contributions from the nitrogen heteroatom in the upper valence region, consistent with the presence of an N-centered lone pair. The density of states calculation confirms that both isomers possess qualitatively similar electronic structures, with occupied orbitals dominating the valence manifold and a nitrogen-based contribution at the HOMO region. The quantitative differences in HOMO energy (−6.24 eV for 1-aza*vs.* −6.35 eV for 9-aza) reflect subtle differences in the electronic environment of the nitrogen heteroatom, ultimately manifesting in differential binding affinities for coordinated cations (Table 1). In contrast, the DOS of 9-azahomocubane displays sharper, more discrete features, suggesting more localized frontier orbitals and a weaker perturbation of the carbon cage electronic structure. These distinctions reflect the different ways in which substitution at the 1- *versus* 9-position breaks the parent homocubane symmetry and perturbs the electronic degeneracy of the cage. The frontier electronic region also highlights notable contrasts between the two isomers. The HOMO of 1-azahomocubane is both higher in energy and a bit delocalized than that of 9-azahomocubane, while the LUMO states in both cases are observed on carbon antibonding orbitals. As a result, the HOMO–LUMO gap is slightly narrower in the 1-substituted isomer, implying enhanced polarizability and a lower energetic barrier for electronic excitation. These modest differences suggest slight variations in electronic softness and polarizability, but the overall electronic structures of the two isomers remain closely comparable. Moreover, the HOMO → LUMO transitions in 1-azahomocubane are expected to carry greater oscillator strength due to improved orbital overlap, providing a rationale for potential differences in their UV-vis absorption profiles. Collectively, the DOS analysis establishes that positional substitution of the nitrogen atom exerts an influence on the electronic structure, with direct implications for the spectroscopic and redox behavior of these strained heterocages.

**Table 1:** Calculated energy of HOMO (*E*_HOMO_, eV), energy of LUMO (*E*_LUMO_, eV), HOMO–LUMO energy gap (*E*_H–L_, eV), Hirshfeld charge on H^+^/M^*n*+^ (HC on H^+^/M^*n*+^), cation affinity (CA, kcal mol^−1^), and cation basicity (CB, kcal mol^−1^) of 1-azahomocubane and 9-azahomocubane and their complexes with H^+^, Li^+^, Na^+^, K^+^, Mg^2+^, and Ca^2+^

Compound	*E*_HOMO_	*E*_LUMO_	*E*_H–L_	HC on N	HC on H^+^/M^*n*+^	CA	CB
1-aza	−6.24	−0.23	6.01	−0.134	—	—	—
(1-aza + H)^+^	−12.31	−4.68	7.63	0.067	0.176	233.63	225.99
(1-aza + Li)^+^	−11.12	−5.23	5.89	−0.141	0.666	48.84	41.50
(1-aza + Na)^+^	−10.70	−5.55	5.15	−0.128	0.732	34.30	27.28
(1-aza + K)^+^	−10.29	−4.89	5.40	−0.136	0.783	23.47	16.99
(1-aza + Mg)^2+^	−15.34	−13.52	1.82	−0.079	1.259	140.66	133.06
(1-aza + Ca)^2+^	−14.73	−11.41	3.32	−0.130	1.416	95.25	88.39
9-aza	−6.35	−0.27	6.08	−0.163	—	—	—
(9-aza + H)^+^	−11.85	−4.83	7.02	0.058	0.182	228.07	220.47
(9-aza + Li)^+^	−10.95	−5.29	5.66	−0.158	0.663	46.13	38.91
(9-aza + Na)^+^	−10.72	−5.58	5.14	−0.147	0.722	32.44	25.58
(9-aza + K)^+^	−10.47	−4.92	5.55	−0.157	0.772	21.88	15.56
(9-aza + Mg)^2+^	−16.03	−12.87	3.16	−0.083	1.094	141.14	132.94
(9-aza + Ca)^2+^	−14.73	−11.41	3.32	−0.130	1.416	97.84	89.81

**Fig. 4:**
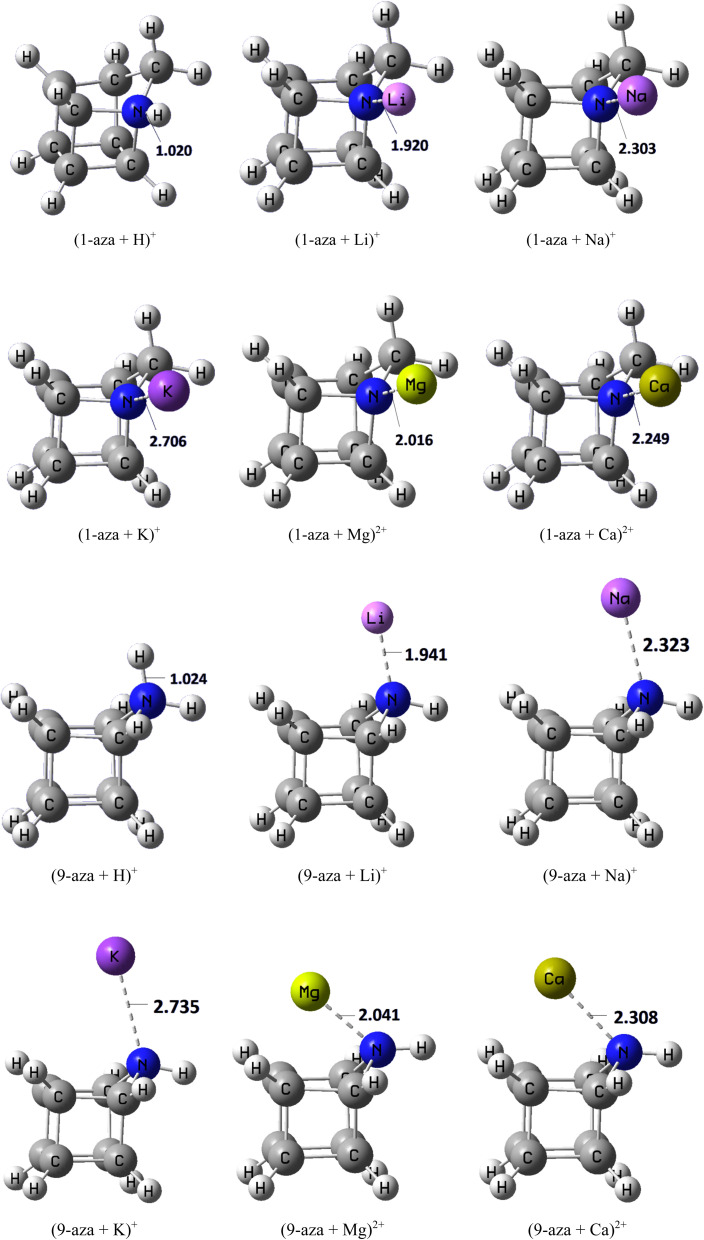
Optimized chemical structures of 1-azahomocubane and 9-azahomocubane complexes with H^+^, Li^+^, Na^+^, K^+^, Mg^2+^, and Ca^2+^.

The Fig. 4 illustrates the optimized geometries of the protonated and metal-coordinated adducts of 1-azahomocubane and 9-azahomocubane, with the corresponding cation–nitrogen bond lengths (in Å) explicitly reported. These distances serve as a direct measure of the strength and nature of the interaction between the heteroatom lone pair and the incoming cation. For protonation (H^+^), the N–H^+^ bond distances are short (≈1.0–1.1 Å, as depicted), reflecting the strong covalent nature of the interaction. Both 1-aza and 9-aza exhibit nearly equivalent bond lengths, consistent with the high proton affinity of the nitrogen site in either cage position. For the alkali metal cations (Li^+^, Na^+^, K^+^), the figure shows a systematic increase in the N–M^+^ bond distance with increasing ionic radius. In the case of Li^+^, the N–Li separation is the shortest (≈1.9–2.0 Å), indicating strong electrostatic attraction due to the high charge density of the small cation. As the cation becomes larger, the distances elongate: N–Na (≈2.3–2.4 Å) and N–K (≈2.7–3.0 Å), in agreement with weaker binding for the more diffuse alkali ions. Notably, for each cation, the bond distances in 1-aza are slightly shorter than those in 9-aza, reflecting the greater accessibility and reduced steric hindrance of the bridgehead nitrogen in the 1-substituted system. For the alkaline-earth dications (Mg^2+^, Ca^2+^), the figure displays significantly shorter N–M^2+^ distances than for the alkali metals of comparable size. This trend reflects the stronger CAs of the divalent cations. The N–Mg^2+^ distance (≈2.0 Å) is particularly short, underscoring the high binding affinity, whereas the N–Ca^2+^ separation (≈2.3–2.4 Å) is somewhat longer but still shorter than the analogous Na^+^ distance, consistent with the doubled charge compensating for the larger radius. A consistent feature throughout the figure is that 1-aza binds all cations at slightly shorter distances than 9-aza. This structural effect can be attributed to the different topological environments of the nitrogen atoms: in 1-aza, the bridgehead position provides more favorable geometry for direct approach of the cation, while in 9-aza, steric and electronic constraints force the cation to remain slightly more distant.

The electronic descriptors collected in Table 1 provide a quantitative basis for understanding how protonation and metal coordination modulate the properties of 1-azahomocubane and 9-azahomocubane. Both neutral cages display HOMO–LUMO gaps of approximately 6.0 eV, consistent with the inherent electronic stability of their strained frameworks. The HOMO of 1-aza is slightly higher in energy (−6.24 eV) than that of 9-aza (−6.35 eV), reflecting a more accessible bridgehead lone pair, while the LUMO of 9-aza is slightly stabilized (−0.27 eV) compared to that of 1-aza (−0.23 eV), indicating a bit higher intrinsic electron affinity at the peripheral site. These subtle baseline differences are amplified upon interaction with protons and cations. Protonation produces the most localized and intense donor–acceptor interaction within the series. For (1-aza + H)^+^, the HOMO is strongly stabilized (−12.31 eV), and the HOMO–LUMO gap expands to 7.63 eV. A similar, though slightly smaller, effect is observed in (9-aza + H)^+^ (7.02 eV). This increase in gap signifies electronic hardening, whereby the system becomes less polarizable and more resistant to further excitation. The charge distribution confirms significant transfer from nitrogen to the bound proton, which carries a positive charge of +0.176*e* in (1-aza + H)^+^ and +0.182*e* in (9-aza + H)^+^. Correspondingly, the CAs are extremely large, exceeding 220 kcal mol^−1^, confirming the exceptional stability of the N–H^+^ interaction. Notably, the bridgehead nitrogen of 1-aza anchors the proton more effectively, consistent with its more localized HOMO. In contrast, binding of alkali cations softens the electronic structure. For 1-aza, the HOMO–LUMO gap decreases to 5.89 eV with Li^+^, 5.15 eV with Na^+^, and 5.40 eV with K^+^. The same pattern is observed in 9-aza (5.66, 5.14, and 5.55 eV, respectively). These reductions in gap indicate increased reactivity and diminished hardness relative to the neutral cages. The extent of charge transfer to the cation follows the expected periodic trend: +0.666*e* on Li^+^, +0.732*e* on Na^+^, and +0.783*e* on K^+^ in the 1-aza series, with nearly identical values in the 9-aza complexes. The CAs decrease markedly with increasing ionic radius, from nearly 49 kcal mol^−1^ for Li^+^ to only 23 kcal mol^−1^ for K^+^ in 1-aza, and from 46 to 22 kcal mol^−1^ in 9-aza. These results highlight the strong dependence of interaction strength on charge density: small, hard Li^+^ ions form stronger contacts, while larger, softer K^+^ ions interact more diffusely and weakly. Importantly, 1-aza exhibits stronger binding and more pronounced softening than 9-aza, again underscoring the dominance of the bridgehead nitrogen. The most dramatic transformations arise upon coordination of divalent cations. In (1-aza + Mg)^2+^, the HOMO and LUMO are both heavily stabilized (−15.34 and −13.52 eV, respectively), collapsing the HOMO–LUMO gap to just 1.82 eV—the narrowest gap in the entire dataset. Such an extraordinarily small gap signifies extreme electronic softness and heightened susceptibility to redox perturbation. The charge distribution confirms strong donor–acceptor interaction, with +1.259*e* localized on Mg^2+^ and nitrogen correspondingly depleted. The CA is very large (≈141 kcal mol^−1^), underscoring the strength of the interaction. The Ca^2+^ complex of 1-aza exhibits a similar but less extreme profile, with a gap of 3.32 eV, a metal charge of +1.416*e*, and CA of ≈95 kcal mol^−1^. In 9-aza, divalent binding is again significant but less pronounced: Mg^2+^ reduces the gap to 3.16 eV, while Ca^2+^ yields 3.32 eV. CAs are comparable to those of 1-aza, but the smaller degree of gap collapse indicates that the peripheral nitrogen engages less strongly in orbital overlap with the metal center. Together, these results establish a clear hierarchy of interaction strength and electronic response. Protonation yields localized, exceptionally stable complexes with increased hardness and expanded HOMO–LUMO gaps. Alkali metalation produces softer, more reactive systems, with binding strength decreasing as ionic radius increases. Alkaline-earth metalation induces the slightly stronger reorganization of the electronic structure, especially in the case of (1-aza + Ca)^2+^, which exhibits extreme gap collapse and significant charge transfer. In many cases, particularly for protonation and alkali metal coordination, 1-azahomocubane exhibits slightly stronger binding than the 9-isomer. However, this trend is not universal, and for alkaline-earth dications—especially Ca^2+^—the binding strengths of the two isomers become comparable or favor 9-azahomocubane. For Mg^2+^ and Ca^2+^, the increased ionic size and polarizability, along with spatial compatibility with the nitrogen cage environment in 9-azahomocubane, facilitate stronger binding. In particular, Ca^2+^ benefits from enhanced overlap and reduced strain within the 9-aza framework, resulting in greater binding affinity compared to its 1-aza analog. This analysis confirms that the positional isomerism of nitrogen fundamentally governs cation binding in azahomocubanes: 1-aza acts as a highly responsive donor center, particularly effective at stabilizing small, hard cations, while 9-aza provides a more diffuse, less perturbative binding site.

**Fig. 5:**
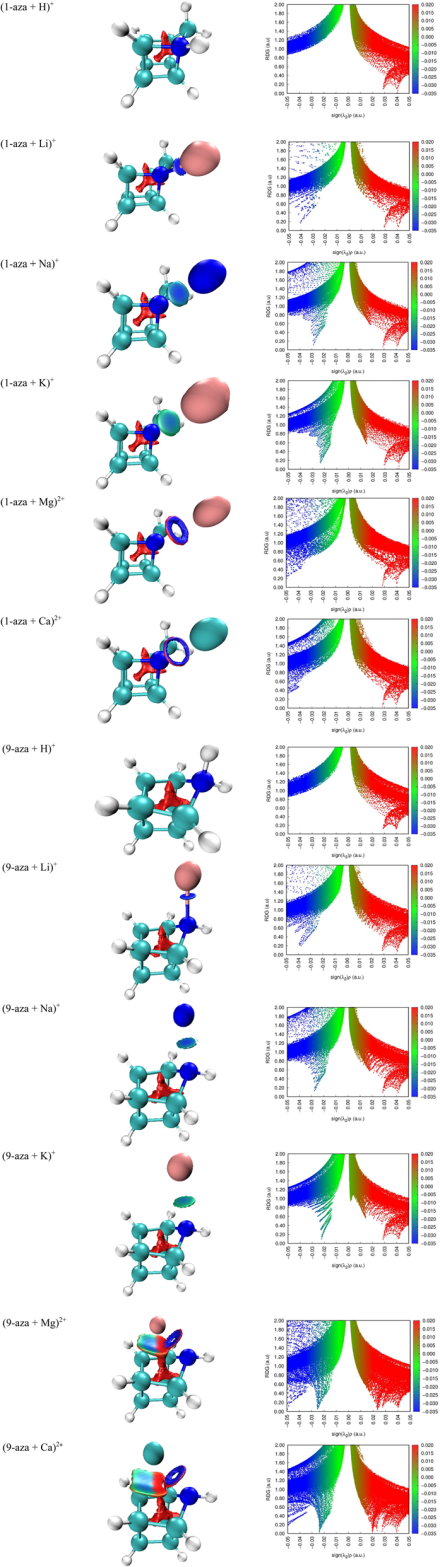
The noncovalent interaction (NCI) surfaces and the reduced density gradient (RDG) scatterplots of complexes of the 1-azahomocubane and 9-azahomocubane with H^+^, Li^+^, Na^+^, K^+^, Mg^2+^, and Ca^2+^.

The noncovalent interaction (NCI) surfaces and the corresponding reduced density gradient (RDG) scatterplots provide a clear and consistent picture of the interaction patterns governing the complexes of the aza-isomers with H^+^, Li^+^, Na^+^, K^+^, Mg^2+^, and Ca^2+^ (Fig. 5). In the protonated species, the NCI isosurfaces display a compact and intensely blue region located along the N–H bond axis, reflecting the formation of a strongly attractive and largely covalent interaction, while the RDG profile is characterized by a sharp, dominant spike at negative values of sign (*λ*_2_)*ρ*, which is diagnostic of strong bonding. Peripheral green patches in the NCI maps and modest density near sign (*λ*_2_)*ρ* ≈ 0 in the RDG plots correspond to weak dispersive contacts involving neighboring CH groups, whereas small red lobes near the N–H environment highlight nonbonding interactions caused by proton attachment. For the alkali cation adducts, the NCI plots show a transition from strongly localized blue-green basins in the Li^+^ complexes to broader and less intense attractive regions in the Na^+^ and K^+^ analogues, indicating a progressive weakening and delocalization of the interaction as the cation size increases and its charge density decreases. In the NCI framework, red regions correspond to nonbonding interactions rather than steric effects *per se*. The presence and extent of these regions reflect areas of unfavorable density overlap and should be interpreted qualitatively. Given the absence of a quantitative color scale, the observed differences between the two isomers are subtle and are best regarded as supportive of trends already established by geometric and energetic analyses. This trend is mirrored in the RDG fingerprints, where Li^+^ generates a pronounced and sharply negative peak, while Na^+^ and K^+^ show reduced peak intensity, a shift toward less negative sign (*λ*_2_)*ρ* values, and increased contributions around sign (*λ*_2_)*ρ* ≈ 0, consistent with a growing role of electrostatics and dispersion. A positive sign (*λ*_2_)*ρ* in the context of ring or cage critical points indicates nonbonded interactions, consistent with excess kinetic energy at these locations. The alkaline-earth metal dications produce the most complex interaction signatures. For both Mg^2+^ and Ca^2+^, the NCI surfaces reveal compact blue basins around the N⋯M^2+^ contacts, reflecting strong attractive interactions enhanced by polarization and partial charge transfer, accompanied by extended green shells between the cation and nearby CH or CC regions that signal ion-induced dispersion and multi-center stabilization. The RDG scatterplots reinforce this picture, displaying an intense negative-sign (*λ*_2_)*ρ* spike corresponding to the direct N⋯M^2+^ interaction, along with a broad distribution of points around sign (*λ*_2_)*ρ* ≈ 0 arising from dispersive stabilization across the cage surface. Compared to Mg^2+^, Ca^2+^ produces slightly less negative main peak positions, together with more pronounced near-zero distributions and enlarged red regions in the NCI maps, all of which point to softer, more diffuse polarization combined with increased nonbonding interactions in accommodating the larger cation. A direct comparison of the two aza-isomers reveals that the position of the nitrogen atom and its steric environment exert a decisive influence on the binding patterns. The isomer in which the nitrogen site is more readily accessible exhibits more concentrated and intensely blue NCI basins at the site of cation coordination, together with stronger and more negative RDG spikes that confirm deeper attractive interactions. In contrast, the sterically encaged nitrogen site gives rise to attenuated blue regions, larger red lobes, and a redistribution of RDG intensity toward values near or above zero, reflecting a greater contribution from dispersion and nonbonding interactions rather than localized attraction. This effect is especially pronounced in the complexes with Na^+^ and K^+^, where the size of the cation accentuates steric constraints, and in the interactions with Mg^2+^ and Ca^2+^, where the balance between strong polarization and steric crowding becomes critical. Overall, the combined NCI and RDG analyses establish a coherent mechanistic interpretation of the binding behavior across the series. Protonation produces a genuinely covalent N–H interaction with minimal secondary contacts, Li^+^ forms a relatively focused and partially covalent ion–dipole interaction at nitrogen, and the larger alkali cations rely increasingly on diffuse electrostatics and dispersion, with steric penalties evident for K^+^. The dications induce strong polarization and multi-center attraction, but with rising nonbonding interactions costs as the ion size increases. The steric accessibility of the nitrogen site in the two isomers ultimately dictates the strength and character of these interactions, with the more accessible nitrogen supporting stronger and more localized binding, while the hindered site enforces weaker, more diffuse, and sterically constrained complexes.

## Conclusions

This systematic computational study establishes a clear structure–property relationship between aza-homocubane topology and cation-binding behavior. Both 1-aza and 9-aza exhibit strong proton affinity, as reflected in short N–H^+^ distances (∼1.0 Å) and highly localized NCI features. For alkali metals, the N–M^+^ separations increase with ionic size (Li^+^ ≈ 1.9 Å, Na^+^ ≈ 2.3 Å, K^+^ ≈ 2.7–2.8 Å), while alkaline earth dications exhibit much shorter binding than comparable monocations (N–Mg^2+^ ≈ 2.0 Å *vs.* N–Na ≈ 2.3 Å), highlighting the influence of charge density. Hirshfeld analysis confirmed the greater extent of charge redistribution in 1-aza complexes: for example, the N charge shifted from −0.134*e* in free 1-aza to +0.732*e* in (1-aza + Na)^+^, compared with −0.163*e* to +0.722*e* in the corresponding 9-aza complex. The CA/CB indices followed the same trend: 34.30/27.28 kcal mol^−1^ for (1-aza + Na)^+^*versus* 32.44/25.58 kcal mol^−1^ for (9-aza + Na)^+^, and 95.25/88.39 kcal mol^−1^ for (1-aza + Ca^2+^)*versus* 97.84/89.81 kcal mol^−1^ for (9-aza + Ca^2+^). In most cases, especially with proton and alkali cations, 1-azahomocubane exhibits stronger binding than the 9-isomer. However, for alkaline-earth dications, notably Ca^2+^, 9-azahomocubane binds more strongly. The NCI isosurfaces and RDG plots further substantiated these conclusions, showing sharper, more localized peaks for proton and Li^+^ interactions in 1-aza, contrasted with broader, weaker features in 9-aza. For Mg^2+^ and Ca^2+^, the RDG minima and dense NCI regions illustrated the highly electrostatic nature of the binding, with enhanced stabilization in 1-aza complexes. Overall, this study demonstrates that positional isomerism of nitrogen in azahomocubanes leads to subtle but reproducible differences in cation binding behavior. While 1-azahomocubane often exhibits slightly shorter interaction distances and marginally enhanced stabilization for proton and alkali metal binding, these effects are quantitatively small, and exceptions arise for alkaline-earth dications, particularly Ca^2+^. The results highlight how cage topology can fine-tune, rather than dominate, ion–ligand interactions in rigid nitrogen-containing frameworks.

## Conflicts of interest

The authors declare no competing interests.

## Data Availability

All data generated or analyzed during this study are included in this published article.
